# Commentary: Pitfalls in the Neuroimaging of Glioblastoma in the Era of Antiangiogenic and Immuno/Targeted Therapy

**DOI:** 10.3389/fneur.2018.00051

**Published:** 2018-02-05

**Authors:** Aaron D. Skolnik, Sumei Wang, Pallavi P. Gopal, Suyash Mohan

**Affiliations:** ^1^Radiology, Penn Medicine Princeton Health, Plainsboro, NJ, United States; ^2^Neuroradiology, Hospital of the University of Pennsylvania, Philadelphia, PA, United States; ^3^Pathology, Yale School of Medicine, New Haven, CT, United States

**Keywords:** glioblastoma multiforme (GBM), MRI, diffusion magnetic resonance imaging, antiangiogenic therapy, targeted therapy, tumor-treating fields

The profoundly aggressive nature of glioblastoma multiforme (GBM) leads to a dismal prognosis, with an overall survival of 15 months with standard surgery and chemoradiation (1, 2). Innovative therapeutic approaches are required to make meaningful survival advances. Therefore, efforts are underway to harness the immune system, target molecular signaling pathways, and even inhibit cell division utilizing alternating electric fields. These new therapies impact the follow-up neuroimaging in ways we are just beginning to understand. Immunotherapies, targeted therapies, antiangiogenic therapy, and tumor-treating fields (TTFields) are discussed, with a brief review of existing challenges in response assessment for these patients, along with some potential solutions.

The evaluation and optimization of novel techniques affecting the tumor microenvironment and signaling is under active investigation, including redirected T lymphocytes (chimeric antigen receptor T-cell, CAR-T), immune checkpoint inhibitors (nivolumab), growth factor/receptor inhibitors and vaccines (i.e., rindopepimut, dendritic cell vaccines), oncolytic virotherapy (i.e., poliovirus), among others (3–7). The harnessing of immune response involves inflammatory sequela which complicates the appearance on neuroimaging. Recognition of these factors has influenced the refinement of response assessment criteria as reflected in the immunotherapy Response Assessment in Neuro-Oncology, with lengthening of the expected window of pseudoprogression from 3 to 6 months, and extending the follow-up interval to confirm radiographic progression from 4 weeks to 3 months (8).

There is a paucity of data evaluating physiologic and metabolic imaging parameters in these patients, necessitating more studies to maximize the potential of advanced imaging tools in detecting elusive disease and redefining response in these patients.

Diffusion weighted imaging (DWI) utilizing apparent diffusion coefficient (ADC) has shown value in immunotherapy-treated GBM. Specifically, minimum ADC values from enhancing areas could differentiate between inflammation and progressive tumor in dendritic cell immunotherapy patients (9). In patients treated with anti-programmed cell death (PD-1) agents (nivolumab and pembrolizumab), after an initial 6-month period of suspected inflammatory hypercellularity, stabilization, and decrease in volumes of intermediate ADC areas correlated with response (10). Diffusion tensor imaging metrics such as fractional anisotropy (FA), linear, planar, and spherical anisotropy coefficients (CL, CP, and CS, respectively) have been shown to characterize tumor microenvironments at the cellular and subcellular level (11–13). These techniques were applied to GBM following standard therapy, and a combination of FA, CL, and maximum relative cerebral blood volume (rCBVmax) had the highest accuracy in identifying true progression (area under the curve 0.91) (12). This multiparametric analysis may allow more accurate assessment following immunotherapy compared to conventional imaging (Figure 1). Perhaps in the future, techniques such as diffusion kurtosis imaging, diffusion spectrum imaging and restriction spectrum imaging (RSI) may allow even further characterization of subtleties of response assessment (14–18).

**Figure 1:**
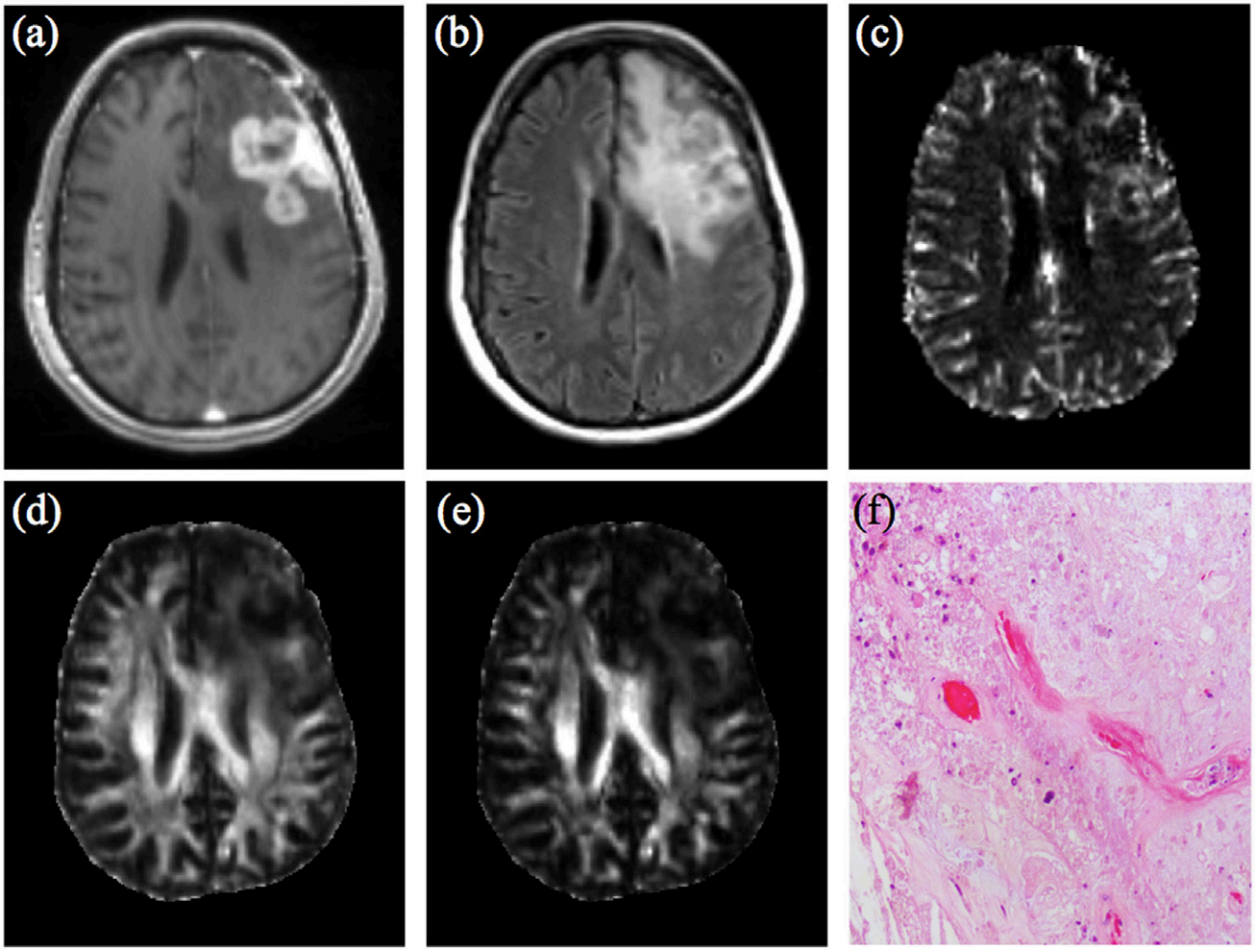
47-year-old woman with GBM, status post gross total resection and chemoradiation, treated with dendritic cell vaccine immunotherapy (ICT-107) (four vaccine treatments over 2 months prior to this imaging.) **(A)** Contrast-enhanced T1-weighted image shows large lobulated nodular enhancing lesion measuring 4.5 cm × 2.8 cm at site of previously resected GBM which had increased from prior scans. **(B)** FLAIR images demonstrate a large area of associated T2/FLAIR signal abnormality in the left hemisphere. **(C)** DSC shows elevated rCBV from the enhancing region of the tumor. Overall constellation of these conventional and advanced imaging findings were concerning for true progression. Logistic regression model combining rCBVmax with FA **(D)** and CL **(E)** according to analysis used in Wang et al. AJNR 2016 did not meet criteria for true progression (rCBVmax 4.396, FA 0.112, CL 0.0418) (12), suggesting a significant component of treatment-related changes. However immunotherapy was discontinued due to concern for progression. **(F)** Pathology from surgical resection performed 2 weeks later demonstrates predominant treatment effect (~80%) with hyalinization of vessels and tissues, geographic necrosis, and macrophage infiltration. Recurrent infiltrating glial tumor cells with marked nuclear pleomorphism were also present, comprising approximately 20% of the specimen. Abbreviations: GBM, glioblastoma multiforme; FLAIR, fluid attenuation inversion recovery; DSC, dynamic susceptibility contrast; rCBV, relative cerebral blood volume; rCBVmax, maximum relative cerebral blood volume; FA, fractional anisotropy; CL, linear anisotropy coefficient.

The targeted anti-vascular endothelial growth factor antibody bevacizumab has drastic effects on tumor vasculature and subsequent imaging. Diffusion has been widely studied in the evaluation of bevacizumab-treated tumors with somewhat complex results, though overall a trend demonstrating increasing restricted diffusion corresponds with worse prognosis in this setting (19–22). RSI has been shown to be less affected by bevacizumab-related changes in T2/FLAIR signal compared to standard DWI and may add specificity to the response assessment following antiangiogenic therapy (18).

Magnetic resonance spectroscopy (MRS) can also add utility in the response assessment of these patients. MRS has been evaluated to assess response to bevacizumab, differentiation of pseudoprogression from true progression, and the genetic profiling of gliomas (23–25). Elevated lipid and low choline/*N*-acetyl aspartate (NAA) ratios have been reported in association with pseudoprogression (24, 26). Early experience with whole-brain echo-planar spectroscopic imaging following standard therapy also shows higher Cho/Cr and Cho/NAA ratios in patients with true progression compared to pseudoprogression, with further improved discriminatory accuracy with multivariate logistic regression analyses (27). The added value of MRS in the immunotherapy setting is yet to be determined.

Tumor-treating fields utilize alternating electric fields to inhibit mitosis (28, 29) and was FDA approved for recurrent GBM in 2011 and for newly diagnosed GBM in 2015 (30, 31). Initial experience utilizing advanced imaging techniques demonstrates decreased FA, Cho/Cr ratio, and rCBVmax, along with increased ADC within the first 2 months in patients treated by TTFields (32, 33). Understanding the imaging findings in patients treated with TTFields requires further investigation, to see if and how the appearance and timing of pseudoprogression and true progression in these patients differ from tumors treated with standard therapy and immunotherapy.

The devastating prognosis of GBM which has only been modestly improved by recent efforts is a testament to the exceedingly adaptive behavior of this tumor, with the best currently available overall survival of 20.5 months with TTFields in addition to standard therapy (31). As a result, the evolving standard of care will likely require a multimodality approach incorporating TTFields and immunotherapy with surgery, radiation, and chemotherapy, the optimal combination of which is still being defined. These new therapies have the capability to mask and/or mimic disease on conventional images and, therefore, imaging evaluation is challenging. Advanced MR techniques have shown potential to further differentiate true progression from pseudoprogression, though efforts to understand these subtleties are early in development. There are barriers to adopt these imaging tools into clinical workflow, including added time for scanning and processing data, which often requires subspecialized knowledge and/or software that is not readily available. Optimizing understanding and use of readily available imaging data such as ADC values may provide added value without added acquisition or processing time. Efforts should also be made to create user friendly applications to process and interpret advanced MRI data. Furthermore, computation of multiparametric radiomic data may allow distillation of many imaging variables into a clinically relevant synthesis, potentially aiding in response assessment (34). Close collaboration with neuropathology, neurosurgery, and neuro-oncology is also critical in the optimization of response assessment. For example, the quantification of a pathologic specimen’s proportion of tumor vs. treatment effects [i.e., the histologic analysis used in Wang et al. (12)], and the use of image-matched specimens can further elucidate tissue composition and define treatment response. Continued multidisciplinary efforts are necessary to better define treatment response and guide therapy in patients with GBM.

## Author Contributions

AS: first author; SW: MRI image processing, figure, and technical input; PG: neuropathology and figure input; and SM: senior author.

## Conflict of Interest Statement

The authors declare that the research was conducted in the absence of any commercial or financial relationships that could be construed as a potential conflict of interest.

## References

[1] Huang RY Neagu MR Reardon DA Wen PY. Pitfalls in the neuroimaging of glioblastoma in the era of antiangiogenic and immuno/targeted therapy – detecting illusive disease, defining response. Front Neurol (2015) 6:33. 10.3389/fneur.2015.00033 25755649 PMC4337341

[2] Stupp R Mason WP van den Bent MJ Weller M Fisher B Taphoorn MJ Radiotherapy plus concomitant and adjuvant temozolomide for glioblastoma. N Engl J Med (2005) 352(10):987–96. 10.1056/NEJMoa043330 15758009

[3] Codrici E Enciu A Popescu I Mihai S Tanase C. Glioma stem cells and their microenvironments : providers of challenging therapeutic targets. Stem Cells Int (2016) 2016:5728438. 10.1155/2016/5728438 26977157 PMC4764748

[4] Cruceru ML Enciu AM Popa AC Albulescu R Neagu M Tanase CP Signal transduction molecule patterns indicating potential glioblastoma therapy approaches. Onco Targets Ther (2013) 9(6):1737–49. 10.2147/OTT.S52365 24348050 PMC3848931

[5] Brown CE Alizadeh D Starr R Weng L Wagner JR Naranjo A Regression of glioblastoma after chimeric antigen receptor T-cell therapy. N Engl J Med (2016) 375(26):2561–9. 10.1056/NEJMoa1610497 28029927 PMC5390684

[6] Polivka J Jr Polivka J Holubec L Kubikova T Priban V Hes O Advances in experimental targeted therapy and immunotherapy for patients with glioblastoma multiforme. Anticancer Res (2017) 37(1):21–33. 10.21873/anticanres.11285 28011470

[7] Brown MC Gromeier M. Oncolytic immunotherapy through tumor-specific translation and cytotoxicity of poliovirus. Discov Med (2015) 19(106):359–65. 26105699 PMC4780852

[8] Okada H Weller M Huang R Finocchiaro G Gilbert MR Wick W Immunotherapy response assessment in neuro-oncology: a report of the RANO working group. Lancet Oncol (2015) 16(15):e534–42. 10.1016/S1470-2045(15)00088-1 26545842 PMC4638131

[9] Vrabec M Van Cauter S Himmelreich U Van Gool SW Sunaert S De Vleeschouwer S MR perfusion and diffusion imaging in the follow-up of recurrent glioblastoma treated with dendritic cell immunotherapy: a pilot study. Neuroradiology (2011) 53(10):721–31. 10.1007/s00234-010-0802-6 21107549

[10] Qin L Li X Stroiney A Qu J Helgager J Reardon DA Advanced MRI assessment to predict benefit of anti-programmed cell death 1 protein immunotherapy response in patients with recurrent glioblastoma. Neuroradiology (2017) 59(2):135–45. 10.1007/s00234-016-1769-8 28070598 PMC6097616

[11] Wang S Kim S Zhang Y Wang L Lee EB Syre P Determination of grade and subtype of meningiomas by using histogram analysis of diffusion-tensor imaging metrics 1. Radiology (2012) 262(2):584–92. 10.1148/radiol.11110576 22084207

[12] Wang S Martinez-Lage M Sakai Y Chawla S Kim SG Alonso-Basanta M Differentiating tumor progression from pseudoprogression in patients with glioblastomas using diffusion tensor imaging and dynamic susceptibility contrast MRI. AJNR Am J Neuroradiol (2016) 37(1):28–36. 10.3174/ajnr.A4474 26450533 PMC7960225

[13] Agarwal A Kumar S Narang J Schultz L Mikkelsen T Wang S Morphologic MRI features, diffusion tensor imaging and radiation dosimetric analysis to differentiate pseudo-progression from early tumor progression. J Neurooncol (2013) 112(3):413–20. 10.1007/s11060-013-1070-1 23417357

[14] Steven AJ Zhuo J Melhem ER. Diffusion kurtosis imaging: an emerging technique for evaluating the microstructural environment of the brain. AJR Am J Roentgenol (2014) 202(1):W26–33. 10.2214/AJR.13.11365 24370162

[15] Wu EX Cheung MM MR diffusion kurtosis imaging for neural tissue characterization. NMR Biomed (2010) 23(7):863–848. 10.1002/nbm.1506 20623793

[16] Wedeen VJ Wang RP Schmahmann JD Benner T Tseng WY Dai G Diffusion spectrum magnetic resonance imaging (DSI) tractography of crossing fibers. Neuroimage (2008) 41(4):1267–77. 10.1016/j.neuroimage.2008.03.036 18495497

[17] White NS McDonald C Farid N Kuperman J Karow D Schenker-Ahmed NM Diffusion-weighted imaging in cancer: physical foundations and applications of restriction spectrum imaging. Cancer Res (2014) 74(17):4638–52. 10.1158/0008-5472.CAN-13-3534 25183788 PMC4155409

[18] Kothari P White NS Farid N Chung R Kuperman JM Girard HM Longitudinal restriction spectrum imaging is resistant to pseudoresponse in patients with high-grade gliomas treated with bevacizumab. AJNR Am J Neuroradiol (2013) 34(9):1752–7. 10.3174/ajnr.A3506 23578667 PMC3925350

[19] Wen Q Jalilian L Lupo JM Molinaro AM Chang SM Clarke J Comparison of ADC metrics and their association with outcome for patients with newly diagnosed glioblastoma being treated with radiation therapy, temozolomide, erlotinib and bevacizumab. J Neurooncol (2015) 121(2):331–9. 10.1007/s11060-014-1636-6 25351579 PMC4311062

[20] Barajas RF Jr Butowski NA Phillips JJ Aghi MK Berger MS Chang SM The development of reduced diffusion following bevacizumab therapy identifies regions of recurrent disease in patients with high-grade glioma. Acad Radiol (2016) 23(9):1073–82. 10.1016/j.acra.2016.04.004 27443507 PMC5571825

[21] Nguyen HS Milbach N Hurrell SL Cochran E Connelly J Bovi JA Progressing bevacizumab-induced diffusion restriction is associated with coagulative necrosis surrounded by viable tumor and decreased overall survival in patients with recurrent glioblastoma. AJNR Am J Neuroradiol (2016) 37(12):2201–8. 10.3174/ajnr.A4898 27492073 PMC5161572

[22] Zhang M Gulotta B Thomas A Kaley T Karimi S Gavrilovic I Large-volume low apparent diffusion coefficient lesions predict poor survival in bevacizumab-treated glioblastoma patients. Neuro Oncol (2016) 18(5):735–43. 10.1093/neuonc/nov268 26538618 PMC4827045

[23] Jeon JY Kovanlikaya I Boockvar JA Mao X Shin B K Burkhardt J Metabolic response of glioblastoma to superselective intra-arterial cerebral infusion of bevacizumab : a proton MR spectroscopic. AJNR Am J Neuroradiol (2012) 33(11):2095–102. 10.3174/ajnr.A3091 22576886 PMC7965590

[24] Sawlani V Taylor R Rowley K Redfern R Martin J Poptani H. Magnetic resonance spectroscopy for differentiating pseudo-progression from true progression in GBM on concurrent chemoradiotherapy. Neuroradiol J (2012) 25(5):575–86. 10.1177/197140091202500511 24029093

[25] Pope WB Prins RM Albert Thomas M Nagarajan R Yen KE Bittinger MA Non-invasive detection of 2-hydroxyglutarate and other metabolites in IDH1 mutant glioma patients using magnetic resonance spectroscopy. J Neurooncol (2012) 107(1):197–205. 10.1007/s11060-011-0737-8 22015945 PMC3650613

[26] Zhang H Ma L Wang Q Zheng X Wu C Xu BN. Role of magnetic resonance spectroscopy for the differentiation of recurrent glioma from radiation necrosis: a systematic review and meta-analysis. Eur J Radiol (2014) 83(12):2181–9. 10.1016/j.ejrad.2014.09.018 25452098

[27] Verma G Mohan S Chawla S Whole-brain echo planar spectroscopic imaging distinguishes recurrent tumor versus pseudoprogression in glioblastoma patients. Sci Poster ISMRM (2016).

[28] Kirson ED Gurvich Z Schneiderman R Dekel E Itzhaki A Wasserman Y Disruption of cancer cell replication by alternating electric fields. Cancer Res (2004) 64(9):3288–95. 10.1158/0008-5472.CAN-04-0083 15126372

[29] Kirson ED Dbalý V Tovarys F Vymazal J Soustiel JF Itzhaki A Alternating electric fields arrest cell proliferation in animal tumor models and human brain tumors. Proc Natl Acad Sci U S A (2007) 104(24):10152–7. 10.1073/pnas.0702916104 17551011 PMC1886002

[30] Stupp R Wong ET Kanner AA Steinberg D Engelhard H Heidecke V NovoTTF-100A versus physician’s choice chemotherapy in recurrent glioblastoma: a randomised phase III trial of a novel treatment modality. Eur J Cancer (2012) 48(14):2192–202. 10.1016/j.ejca.2012.04.011 22608262

[31] Stupp R Taillibert S Kanner AA Kesari S Steinberg DM Toms SA Maintenance therapy with tumor-treating fields plus temozolomide vs temozolomide alone for glioblastoma: a randomized clinical trial. JAMA (2015) 314(23):2535–43. 10.1001/jama.2015.16669 26670971

[32] Mohan S Chawla S Wang S Verma G Skolnik A Brem S Assessment of early response to tumor-treating fields in newly diagnosed glioblastoma using physiologic and metabolic MRI: initial experience. CNS Oncol (2016) 5(3):137–44. 10.2217/cns-2016-0003 27076281 PMC6042635

[33] Mohan S Chawla S Skolnik A Poptani H Perspective on the EF-14 trial and its implications for the role of tumor-treating fields in the management of glioblastoma. Transl Cancer Res (2016) 5:Sulement2 10.21037/tcr.2016.07.49

[34] Tiwari P Prasanna P Wolansky L Pinho M Cohen M Nayate AP Computer-extracted texture features to distinguish cerebral radionecrosis from recurrent brain tumors on multiparametric MRI: a feasibility study. AJNR Am J Neuroradiol (2016) 37(12):2231–6. 10.3174/ajnr.A4931 27633806 PMC5161689

